# The Genetic Architecture of Multiple Myeloma

**DOI:** 10.1155/2014/864058

**Published:** 2014-04-03

**Authors:** Steven M. Prideaux, Emma Conway O'Brien, Timothy J. Chevassut

**Affiliations:** Brighton and Sussex Medical School, Sussex University, Falmer, Brighton BN1 9PS, UK

## Abstract

Multiple myeloma is a malignant proliferation of monoclonal plasma cells leading to clinical features that include hypercalcaemia, renal dysfunction, anaemia, and bone disease (frequently referred to by the acronym CRAB) which represent evidence of end organ failure. Recent evidence has revealed myeloma to be a highly heterogeneous disease composed of multiple molecularly-defined subtypes each with varying clinicopathological features and disease outcomes. The major division within myeloma is between hyperdiploid and nonhyperdiploid subtypes. In this division, hyperdiploid myeloma is characterised by trisomies of certain odd numbered chromosomes, namely, 3, 5, 7, 9, 11, 15, 19, and 21 whereas nonhyperdiploid myeloma is characterised by translocations of the immunoglobulin heavy chain alleles at chromosome 14q32 with various partner chromosomes, the most important of which being 4, 6, 11, 16, and 20. Hyperdiploid and nonhyperdiploid changes appear to represent early or even initiating mutagenic events that are subsequently followed by secondary aberrations including copy number abnormalities, additional translocations, mutations, and epigenetic modifications which lead to plasma cell immortalisation and disease progression. The following review provides a comprehensive coverage of the genetic and epigenetic events contributing to the initiation and progression of multiple myeloma and where possible these abnormalities have been linked to disease prognosis.

## 1. Overview of Myeloma Genetics

Myeloma is a genetically complex disease which develops via a multistep process whereby plasma cells are driven towards malignancy through the accumulation of genetic “hits” over time. This multistep process permits myeloma to have various recognisable clinical phases, distinguished by biological parameters, along its development (Table 1). The earliest of these phases is termed monoclonal gammopathy of undetermined significance (MGUS) and is an indolent, asymptomatic, premalignancy phase characterized by a small clonal population of plasma cells within the bone marrow of <10% [1]. MGUS has a prevalence of >5% in adults aged over 70 and a progression risk to myeloma quantified at 1% per year [2, 3]. Following MGUS is smouldering multiple myeloma (SMM), another asymptomatic phase distinguished from MGUS by a greater intramedullary tumour cell content of >10% and an average risk of progression to myeloma of 10% per year for the first five years [4]. Next, myeloma itself is recognised, whereby malignant clones cause clinically relevant end-organ damage including the features of CRAB. The final phase is plasma cell leukemia (PCL), an aggressive disease end-point characterised by the existence of extramedullary clones and rapid progression to death. The basic premise of this disease progression is that the accumulation of genetic “hits” across different cellular pathways drives malignant change through deregulation to the intrinsic biology of the plasma cell. With advancements in molecular biology, many of these disrupted genes and pathways have now been characterised and the current challenge is therefore how to correctly interpret these molecular findings and develop them into clinically useful advances.

### 1.1. Myeloma Intraclonal Heterogeneity

**Figure 1:**
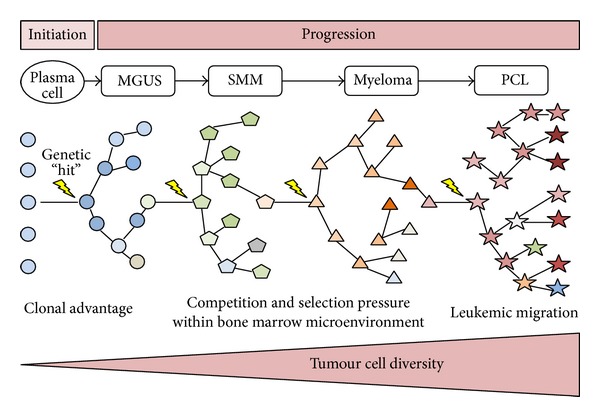
*Initiation and progression of myeloma*. A postgerminal centre B cell receives a genetic “hit” which immortalizes the cell and initiates transition to the indolent phase of monoclonal gammopathy of undetermined significance (MGUS). MGUS clones may then transition through the other disease phases of smouldering multiple myeloma (SMM), myeloma, and plasma cell leukemia (PCL) as genetic “hits”, which confer a survival advantage and are acquired over time. Clonal evolution develops through branching pathways whereby numerous ecosystems composed of multiple subclones exist at each disease phase, as represented by the differing shapes. At the end of this process, proliferative clones no longer become confined to the bone marrow and expand rapidly as a leukemic phase. At each disease phase, the precursor clones are present only at a low level as they have been outcompeted by more advantageous clones. It should be noted that the above figure represents an oversimplification of myeloma initiation and progression, as the process is highly complex with multiple pathways possible at any one time (adapted from Morgan et al., 2012 [8]).

Alongside aiding the characterisation of genes and pathways disrupted in myeloma, molecular studies have also revealed that intraclonal heterogeneity is a common feature of the malignancy [5, 6]. This heterogeneity adds an extra layer of complexity to myeloma progression as it is apparent that genetic “hits” are not acquired in a linear fashion but rather through nonlinear branching pathways synonymous to Darwin's evolution of the species [7, 8]. A model of myeloma development through branching pathways is represented in Figure 1. This model however is designed as an oversimplification and should be viewed as a gross overview of disease progression as the process is highly complex with multiple progression pathways possible [8]. This analogy to Darwin's work explains that plasma cell clones acquire genetic lesions randomly and that these aberrations are then selected out based on their survival advantage. Consideration of intraclonal heterogeneity is important for disease understanding, as it is likely that the findings from many genomic studies represent the genetic aberrations in the predominant clonal population at the time of sampling and that these results may not be applicable to all subclonal populations. This has particular therapeutic relevance, as the genes and pathways deregulated in the predominant clonal population are unlikely to be uniform across the many subclones allowing drug resistance and relapse to occur through the evolution and progression of these minority populations.

### 1.2. Nonhyperdiploid and Hyperdiploid Myeloma

**Figure 2:**
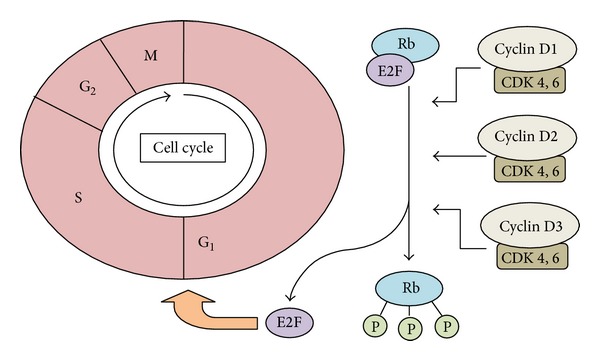
*Overexpression of cyclin D genes influence cell cycle progression at the G*_*1*_ */S transition point in myeloma*. Increased cyclin D gene expression through hyperdiploid or nonhyperdiploid events in myeloma facilitates activation of a cyclin-dependent kinase (CDK 4 or 6). The respective CDK then phosphorylates Rb (retinoblastoma protein), which subsequently resides from its role inhibiting E2F transcription factors allowing these to facilitate cell cycle progression at the G_1_/S transition. G_1_: Gap-1 phase; S: synthesis phase; G2: Gap-2 phase; M: mitosis; P: phosphate group.

Along the progression from MGUS to PCL, genetic aberrations can be classified as primary events, contributing to plasma cell immortalisation, or secondary events, contributing to disease progression. This classification facilitates the division of myeloma into two broad groups, nonhyperdiploidy myeloma and hyperdiploidy myeloma, based on one of two genetic aberrations observed in the primary phase [9, 10], a distinction originally suggested by Smadja et al., supported by the work of others, who put forward the idea of myeloma representing two closely related diseases [11–13]. Nonhyperdiploidy myeloma involves the translocation (*t*) of immunoglobulin heavy chain alleles (IGH@) at 14q32 with various partner chromosomes including 4, 6, 11, 16, and 20. These primary translocations occur due to aberrant class switch recombination (CSR) in lymph node germinal centres and act to juxtapose the partner chromosome oncogenes under the influence of the IGH@ enhancer region. Hyperdiploidy myeloma is generally associated with better survival and involves trisomies of the odd numbered chromosomes 3, 5, 7, 9, 11, 15, 19, and 21 coupled to a low prevalence of IGH@ translocations [14, 15]. Either directly, or indirectly, one consequence of hyperdiploid and nonhyperdiploid events is to result in deregulation of the G_1_/S cell cycle transition point via the overexpression of cyclin D genes, an event shown to be a key early molecular abnormality in myeloma (Figure 2) [16]. For completion, it should be stated that exceptions to the hyperdiploidy and nonhyperdiploidy divisions do exist and that cases with primary translocations and multiple trisomies are detected in a minority.

### 1.3. Secondary Genetic Events and the Bone Marrow Microenvironment

Secondary genetic events drive disease progression and are generally found at higher frequencies in SMM, myeloma, and PCL. These secondary events cooperate with primary events to produce the malignant phenotype of myeloma and include secondary translocations, copy number variations (CNV), loss of heterozygosity (LOH), acquired mutations, and epigenetic modifications. Coupled to the development of these secondary events, clonal cells require a specialised relationship with bone marrow stromal cells for growth and survival. Studies have shown that this microenvironment interaction is highly complex, involving positive and negative interactions between the many cell types mediated through a variety of adhesion molecules, receptors, and cytokines [17, 18]. Furthermore, the derangement of these stromal-clone interactions has been shown to have important consequences in facilitating plasma cell homing to the bone marrow [18], promoting plasma cell immortalisation, and helping spread to secondary bone marrow sites [19, 20]. This stromal-clone relationship is relatively poorly understood at present but represents an area where investigation is ongoing and treatments are likely to be developed [21, 22].

### 1.4. Inherited Variation

Several studies have demonstrated that the majority of, if not all, myeloma cases pass through the MGUS phase [23, 24]. Therefore, in order to gain a fuller understanding of the disease, it is important to consider the genetic and environmental factors influencing transition to this indolent phase. From familial studies on index cases of myeloma, it is apparent that inherited genetic variation can predispose to the development of MGUS as these families have a two- to four-fold increased risk of developing the premalignant condition [25]. By investigating these families further, molecular epidemiology studies identified three genetic loci with associated gene pairs (2p:* DNMT3A* and* DTNB*, 3p:* ULK4* and* TRAK1*, 7p:* DNAH11* and* CDCA7L*) which incur a modest but increased risk of developing myeloma [26]. The complete functional role of these gene pairs is currently unknown, although deregulation of the proto-oncogene* MYC* encoding a transcription factor which regulates genes involved in DNA replication, cell proliferation, and apoptosis, has been implicated [26]. From these initial studies, it is likely that more susceptibility loci will be identified in the future and that these may be correlated to specific myeloma subtypes. The reliable identification of those at risk of developing myeloma would be an important advancement as it may facilitate comprehensive disease monitoring and early disease detection. Furthermore, it could be postulated that future targeted therapies or gene knockdown interventions may be developed against these susceptibility loci to restrict progression to myeloma altogether.

## 2. Chromosomal Translocations in Myeloma

**Figure 3:**
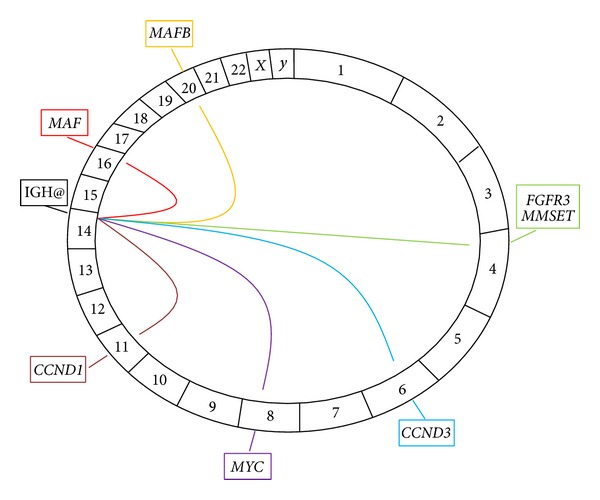
*The key chromosomal translocations in myeloma*. A Circos plot, with the chromosomes arranged in a clockwise direction, demonstrating the key translocations in myeloma. The translocations are represented as lines emerging from the immunoglobulin heavy chain (IGH@) locus on chromosome 14 to their respective partner chromosomes. The genes involved in each translocation are represented in boxes outside the plot. All translocations represent primary events except *t*(8; 14) involving* MYC* which is a secondary translocation.

Chromosomal translocations account for 40–50% of primary events in myeloma and strongly influence disease phenotype [9]. Secondary translocations, not associated with aberrant CSR, occur later in disease and are likely to represent progression events. The key primary and secondary translocations occurring in myeloma are highlighted in Figure 3.

### 2.1. *t*(4; 14) in Myeloma

The *t*(4; 14) is observed in 15% of myeloma cases and has been associated with an adverse prognosis in a variety of clinical settings such as those receiving high dose therapy with autologous stem cell transplant (ASCT) [27–30]. Pathologically, *t*(4; 14) results in the overexpression of two genes,* FGFR3* and* MMSET*, by juxtaposition next to the IGH@ enhancers [31]. The upregulation of* FGFR3* results in the ectopic expression of the FGFR3 tyrosine kinase receptor, an aberration with a currently unclear role in myelomagenesis. Interestingly, the pathogenic role of* FGFR3* is somewhat in question, as approximately 30% of *t*(4; 14) tumours are imbalanced and lack* FGFR3* expression due to loss of the derivative 14 chromosome [28, 32]. Furthermore, in these 30% lacking* FGFR3* expression, the adverse prognosis of *t*(4; 14) remains [28], lending support for the role of the second gene* MMSET*.* MMSET* is overexpressed in all *t*(4; 14) tumours and encodes a chromatin-remodelling factor with histone methyltransferase (HMT) activity [27]. As for* FGFR3*, the exact role* MMSET* plays in pathogenesis is unclear although epigenetic regulation and a role in DNA repair have been suggested [33, 34]. In keeping with the unifying event of cyclin D deregulation, *t*(4; 14) with* MMSET* and/or* FGFR3* overexpression have been shown to upregulate* CCND2*, and in some instances* CCND1*, through an unknown mechanism [9]. It is interesting to note that despite the poor prognosis associated with *t*(4; 14) a clear survival advantage in these tumours has recently been demonstrated through early treatment with the proteasome inhibitor bortezomib [35, 36], with a suggestion that prolonged bortezomib treatment can overcome the adverse prognosis altogether [35, 36]. This point demonstrates that future myeloma prognostication is likely to be determined by the success of therapeutically targeting high-risk lesions through a personalised approach.

### 2.2. *t*(6; 14) and *t*(11; 14) in Myeloma

The *t*(6; 14) is a rare translocation present in 2% of myeloma patients which results in the direct upregulation of the* CCND3* gene via juxtaposition to the IGH@ enhancers [27, 38]. *t*(11; 14) is more common, occurring in approximately 17% of myeloma patients and also directly upregulates a cyclin D gene in the form* CCND1* [27, 39]. Gene expression studies have shown that the overexpression of* CCND3* and* CCND1* results in a clustering of downstream gene expression suggesting that activation of these two genes results in the deregulation of common downstream transcriptional events [27]. Due to the seeming importance of cyclin D gene deregulation in myeloma, cyclin D inhibitors with a variety of specificities have shown promise targeting myeloma* in vitro* [40, 41], with many of these inhibitors now entering early human trials. Unlike *t*(4; 14), the overall prognostic impact of these two translocations is neutral [42], although *t*(11; 14) patients do show considerable heterogeneity and in some instances the translocation may manifest with an aggressive phenotype such as PCL.

### 2.3. *t*(14; 16) and *t*(14; 20) in Myeloma

The *t*(14; 16) and *t*(14; 20) both result in increased expression of a* MAF* family oncogene and combined are identified in 5–10% of presenting myeloma cases [27]. Specifically, *t*(14; 16) results in overexpression of the* MAF* gene splice variant* c-MAF*, a transcription factor which upregulates a number of genes including* CCND2* by binding directly to its promoter [43]. *t*(14; 16) has been associated with a poor prognosis in a number of clinical series [29, 44], although this concept has recently been challenged by retrospective multivariate analysis on 1003 newly diagnosed myeloma patients which showed *t*(14; 16) not to be prognostic [45]. *t*(14; 20) is the rarest translocation involving the IGH@ and results in upregulation of the* MAF* gene paralog* MAFB*. Microarray studies have demonstrated that* MAFB* overexpression results in a very similar gene expression profile (GEP) to that seen with* c-MAF* [27], suggesting that common downstream targets, including* CCND2*, are deregulated by each. Interestingly, *t*(14; 20) is associated with a poor prognosis when present in myeloma but correlates to long-term stable disease when found in MGUS and SMM [46]. This suggests that the translocation alone is not responsible for the poor prognosis but that additional genetic events are required.

### 2.4. Secondary Translocations in Myeloma

As opposed to primary translocations, secondary translocations are CSR-independent events occurring later in disease. Furthermore, although the most frequent secondary translocation is *t*(8; 14), they do not always involve the IGH@ at 14q32 with approximately 40% linking different partner genes [47]. The gene typically deregulated by secondary translocations is* MYC*, the overexpression of which is linked directly to late disease stages and indirectly to a poor prognosis via a strong correlation to high levels of serum *β*_2_-microglobulin (S*β*_2_M) [48], an established indicator of a poor prognosis [49]. The frequency of* MYC* overexpression from secondary translocations supports its role as a progression event, as it is infrequently witnessed in MGUS but seen in 15% of myelomas and 50% of advanced disease [48, 50]. In opposition to this, a mouse model has previously demonstrated that the sporadic activation of a* MYC* transgene in germinal centre B cells of MGUS-prone mice results in the universal development of myeloma [51], whereas as previously discussed, an association also exists between* MYC* deregulation and certain genetic loci linked with myeloma susceptibility [26]. From these conflicting findings, it appears that* MYC* may play a role in both early and late disease phases and that further studies are required to elucidate an exact role for the gene.

## 3. Copy Number Variations in Myeloma

Copy number variations result from gains and losses of DNA and are common events in myeloma. These gains and losses can be both focal or of an entire chromosome/chromosome arm. In general, losses of DNA contribute to malignancy through loss of tumour suppressor genes, whereas gains are pathogenic through oncogene overexpression/activation.

### 3.1. Hyperdiploidy

Hyperdiploidy involves trisomies of the odd numbered chromosomes and is an event witnessed in approximately 50% of myeloma cases [14]. More common in elderly patients and associated with a high incidence of bone disease, hyperdiploidy confers a relatively favourable prognosis in the majority of cases [14], a factor held particularly true in instances where amplification 5q31.3 is concurrently present [52]. The underlying mechanism to generate hyperdiploidy is unknown, although one hypothesis, based on what is suggested to occur in hyperdiploid acute lymphoblastic leukemia, is that a single catastrophic mitosis results in the gain of whole chromosomes rather than their serial accumulation over time [53]. Along with the underlying mechanism, the consequence of hyperdiploidy towards myelomagenesis is poorly understood. However, alongside the known dysfunction of cyclin D genes, recent GEP studies have demonstrated that a high proportion of protein biosynthesis genes, specifically ribosomal protein genes representing end-points in MYC, NF-*κ*B, and MAPK signalling pathways, are also concurrently overexpressed in hyperdiploid tumours [54, 55]. One explanation for this is that these genes are overexpressed due to rapid cell proliferation. This however is unlikely, as myeloma has a distinctively low proliferation rate. Instead, it is proposed that the overexpression is driven by gene copy number increases, with hyperdiploid cells then possessing more ribosomes and translational initiation factors to promote myelomagenesis through the overexpression of cellular growth genes [54].

### 3.2. Gain of 1q

Gain of the chromosome 1q arm (+1q) is an event observed in 35–40% of presenting myeloma cases and one which is frequently observed along with loss of 1p [56–59]. +1q is associated with a poor prognosis in patients treated both intensively and nonintensively and is an observation which remains when other adverse cytogenetic lesions which frequently coexist are removed [57, 60, 61]. Despite this knowledge, the relevant genes on 1q are not fully explored. One region of the chromosome arm which has been identified as a frequently minimally amplified region; however, 1q21 does contain many candidate oncogenes in the form of* CKS1B*,* ANP32E*,* BCL-9*, and* PDZK1* [57, 60, 62]. The importance of this region is supported by the demonstration of a strong association between +1q21 and an adverse prognosis using both fluorescence* in situ* hybridization (FISH) and GEP techniques [56, 57, 61]. Of these genes,* ANP32E*, a protein phosphatise 2A inhibitor with a role in chromatin remodelling and transcriptional regulation, is of particular interest as it has been shown to be independently associated with shortened survival [57]. These findings support the importance of +1q in myeloma pathogenesis and suggest that patients in this group may benefit from specific inhibitors of the candidate genes and pathways identified.

### 3.3. Loss of 1p

Whole arm deletion or interstitial deletions of the 1p chromosome arm are observed in approximately 30% of myeloma patients and are associated with a poor prognosis in a range of treatment settings [57, 63, 64]. Molecular genetics has revealed that two regions of 1p, 1p12, and 1p32.3 are particularly important in myeloma pathogenesis when deleted. 1p12 may be hemi- or homozygously deleted and contains the candidate tumour suppressor gene* FAM46C* [5]. The function of* FAM46C* is unknown, although recent sequencing and homology studies have shown that its expression is correlated to both that of ribosomal proteins and eukaryotic initiation/elongation factors involved in protein translation [5].* FAM46C* is considered a gene of significance as it has been shown to be frequently mutated in myeloma whilst also being independently correlated to a poor prognosis [5, 57, 58, 63]. 1p32.3 may also be hemi- and homozygously deleted and contains the two target genes,* FAF1* and* CDKN2C*.* CDKN2C* is a cyclin-dependent kinase 4 inhibitor involved in negative regulation of the cell cycle, whereas* FAF1* encodes a protein involved in initiation and/or enhancement of apoptosis through the Fas pathway. Homozygous deletion of 1p32.3 is associated with a poor prognosis in those receiving ASCT whereas in those receiving nonintensive treatment its prognostic impact is neutral [63]. Significant evidence points to* CDKN2C* as being the influential gene lost through homozygous 1p32.3 deletion [63, 65], although as* CDKN2C* and* FAF1* lie in such close proximity, the vast majority of deletions lose both genes and therefore the importance of* FAF1* relative to* CDKN2C* is difficult to delineate.

### 3.4. Loss of Chromosome 13/13q

Chromosome 13 deletion is observed in approximately 50% of myeloma cases and is commonly associated with nonhyperdiploid tumours [66–68]. In approximately 85% of cases, deletion of chromosome 13 constitutes a monosomy or loss of the q arm, whereas in the remaining 15% various interstitial deletions occur [66, 69]. With this, the identification of key genes contributing to myeloma pathogenesis is challenging as often a level of gene function remains from the residual allele(s). Despite this, molecular studies have shown that the tumour suppressor gene* RB1* is significantly underexpressed in del(13/13q) and may therefore result in inferior negative cell cycle regulation [57]. To establish the prognostic impact of del(13/13q) is challenging due to its frequent association with other high-risk lesions, such as that of *t*(4; 14) where it is concurrently present in approximately 90% of cases [59]. When del(13/13q) is detected via conventional cytogenetics a link to poor survival exists [70, 71]. However, when detected via FISH, and in the absence of coexisting high-risk lesions, its significance towards survival is lost [42, 72]. This finding suggests that the historical link between del(13/13q) and a poor prognosis is therefore a surrogate of its association with high-risk lesions. One caveat to this statement however is that few long term follow-up studies comparing patient outcomes with or without these high-risk lesions have been completed whereas several long-term studies comparing the presence or absence of del(13/13q) do exist. In one of these studies, conducted by Gahrton et al. [73], a 96-month followup of 357 myeloma patients treated with either autologous transplantation or tandem autologous/reduced intensity conditioning allogenic transplantation (auto/RICallo) showed that whilst del(13/13q) acted as a poor prognostic marker for those receiving autologous transplantation this factor was apparently overcome for patients with del(13/13q) receiving auto/RICallo. This therefore suggests that del(13/13q) may have value as a poor prognostic marker for long-term outcomes in those receiving autologous transplantation.

### 3.5. Loss of 17p

The majority of chromosome 17 deletions are hemizygous and of the whole p arm, a genetic event observed in approximately 10% of new myeloma cases with this frequency increasing in later disease stages [29, 74]. The relevant gene deregulated in del(17p) is thought to be the tumour suppressor gene* TP53*, as GEP has shown that myeloma samples with monoallelic 17p deletions express significantly less* TP53* compared to nondeleted samples [57]. Furthermore, in cases without del(17p) the rate of* TP53* mutation is <1%, whereas in cases with del(17p) this rises to 25–37% [75]; a finding providing some evidence that monoallelic 17p deletion contributes to disruption of the remaining allele. The* TP53* gene has been mapped to 17p13 and is known to function as a transcriptional regulator influencing cell cycle arrest, DNA repair, and apoptosis in response to DNA damage. In myeloma, del(17p) is the most important molecular finding for prognostication as it linked to an aggressive disease phenotype, a greater degree of extramedullary disease, and shortened survival [29, 42, 76]. It is hypothesised that PCL is largely a consequence of* TP53* dysfunction, as the majority of these cases have abnormalities in the gene [74]. Furthermore, most, if not all, human myeloma cell lines which survive in laboratory cell culture have* TP53* deficiency, further suggesting its importance in extramedullary disease. Despite the consensus that* TP53* is the relevant gene disrupted in del(17p); however, it should be stated that no direct biological evidence exists to support this hypothesis and that further exploration of the genetic consequences of the deletion is required.

### 3.6. Other Chromosomal Losses

Many other chromosomal deletions, focal copy number losses, and regions of LOH are seen in myeloma, and as with the deletions of 1p, 13/13q, and 17p, the relatively high frequencies of these events in regions containing tumour suppressor genes suggest they are “driver” lesions contributing to myelomagenesis. Chromosome 11q deletion is observed in 7% of myeloma cases and harbours the tumour suppressor genes* BIRC2* and* BIRC3* [57]. del(14q) is a common event found in 38% of cases and includes the tumour suppressor gene* TRAF3* [57]. 16q deletion is another common event, seen in 35% of myeloma cases, and contains the tumour suppressor genes* CYLD* and* WWOX* [57]. All of these genes, except* WWOX* which is implicated in apoptosis [77], are involved in the NF-*κ*B pathway and demonstrate that activation of this signalling pathway is important in myeloma pathogenesis [57, 78, 79]. del(12p) is another lesion of interest in myeloma, as a recent single-nucleotide polymorphism (SNP) assay found it to be an independent adverse prognostic marker in 192 newly diagnosed patients [52]; a finding however not repeated in other studies [57]. Two further common chromosomal arm deletions frequently witnessed in myeloma are del(6q) and del(8p), observed 33% and 19–24% of cases, respectively [57, 80–82]. The relevance of del(6q) towards survival is as yet not clear. For del(8p) however, it has been shown that this aberration acts as an independently poor prognostic factor for both progression free survival (PFS) and overall survival (OS) [80, 81]. Furthermore, it has been shown that the tumour necrosis factor-related apoptosis-inducing ligand (TRALI) receptor gene is located on 8p, and that during del(8p) a consequential downregulation of TRALI occurs [83]. As TRALI is associated with TNF-induced apoptosis, it is proposed that with reduced receptor expression in del(8p) the sensitivity of tumour cells to TRAIL-medicated apoptosis may be decreased providing an advantage for the immune escape of malignant clones from surveillance by natural killer cells and cytotoxic T lymphocytes [84].

## 4. Deregulation of Myeloma Cellular Pathways and Processes

**Figure 4:**
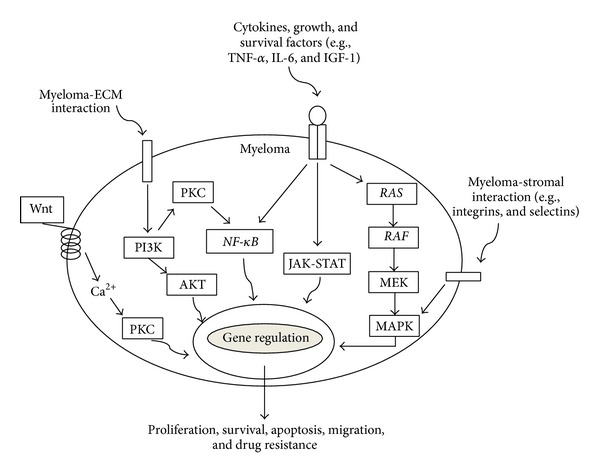
*Signalling pathways involved in myeloma pathogenesis*. The various pathways involved in myeloma pathogenesis may be stimulated via exogenous factors, such as Wnt proteins, myeloma-stromal interactions, cytokines, growth and survival factors, and myeloma-extracellular matrix (ECM) interactions, or the pathways may be aberrantly activated endogenously through genetic abnormalities such as activating mutations in* RAS*,* RAF*, and* NF-*κ*B* genes.

A range of signalling pathways are deregulated in myeloma and contribute towards pathogenesis through associations with proliferation, survival, apoptosis, migration, and drug resistance (Figure 4) [85]. Other cellular processes such as DNA repair, RNA editing, protein homeostasis, and cell differentiation may also contribute towards myelomagenesis through aberrant functioning.

### 4.1. NF-*κ*B

NF-*κ*B comprises a family of structurally related transcription factors which are upregulated during cellular stress to mediate gene responses. Salient to cancer, aberrant activation of NF-*κ*B contributes to cell survival, proliferation and adhesion pathways. In myeloma, the NF-*κ*B pathway is constitutively active in at least 50% of cases and is likely to represent a “driver” event due to its differing activation frequency between MGUS and later disease phases [78, 79]. Interestingly, NF-*κ*B may be upregulated in both plasma cells and surrounding bone marrow stromal cells. In these supporting cells, NF-*κ*B stimulates the release of key cytokines such as IL-6, BAFF and APRIL resulting in paracrine stimulation and critical survival signals to malignant clones [86, 87]. Activation of NF-*κ*B within myeloma cells occurs through a range of mechanisms, including the inactivation of pathway suppressors through gene deletions and/or mutations, and pathway hyperactivity due to translocations and copy number gains [78, 79]. Furthermore, a recent whole genome sequencing (WGS) and whole exome sequencing (WES) study expanded the possible mechanisms through which the pathway may be activated by demonstrating 14 novel mutations/rearrangements affecting 11 NF-*κ*B pathway genes [5]. The frequency with which NF-*κ*B is deregulated in myeloma supports its importance in pathogenesis, although the prognostic impact for many of the implicated genes are yet to be fully elucidated. As the pathway involves the proteasome, inhibitors of this protein complex have been developed, with evidence suggesting that tumours “addicted” to the NF-*κ*B pathway are particularly sensitive to these drugs [79]. Any adverse prognosis of NF-*κ*B activation may therefore be potentially therapeutically ameliorated in the future.

### 4.2. Cell Proliferation

Of the pathways highlighted in Figure 4, three of them, the MAPK pathway, the JAK-STAT pathway, and the PI3K pathway, are particularly implicated in myelomagenesis through influences on cell proliferation.

#### 4.2.1. The Mitogen Activated Protein Kinase (MAPK) Pathway

The MAPK pathway is a highly conserved cellular signalling cascade involved in cell differentiation, proliferation, and survival. The pathway may be stimulated via a range of inflammatory cytokines, such as TNF-a, IL-6, and IGF-1, which in turn activate the downstream kinase cascades RAS, RAF, MEK, and MAPK ultimately influencing gene expression. Two dominant oncogenes in the MAPK pathway, deregulated in many cancers, are* NRAS* and* KRAS*. These genes are frequently mutated in myeloma with a combined prevalence of 20–35% [88].* RAS* mutations are likely to represent progression events as they are rarely found in MGUS but occur more frequently in later disease [89]. Additionally,* RAS* mutations are a poor prognostic marker, frequently being associated to a more aggressive disease phenotype and shortened survival times [88]. Recently however, it has been suggested that* KRAS*, and not* NRAS*, is the more influential gene impacting on prognosis [88], a finding which may have important consequences if genetic lesions are used to define risk. Due to the importance of* RAS* mutations and the MAPK pathway across many cancers, therapeutic inhibitors within this area are a key focus of research.

Showing further importance of the MAPK pathway, a recent study by Chapman et al. identified that seven out of 161 (4%) myeloma patients harboured a previously unobserved mutation in the* BRAF* gene [5].* BRAF* encodes a serine/threonine-protein kinase in which activating mutations are known to be important in many cancers including melanoma and hairy cell leukemia [90]. This has particular clinical relevance, as myeloma patients with* BRAF* mutations may benefit from newly developed BRAF inhibitors, drugs which in some instances have shown marked clinical activity [91]. The premise to perform genome analysis for* BRAF* mutations on 161 samples arose from an original WGS/WES study on 38 myeloma samples which revealed the mutation in one patient [5]. This highlights the advantage of WGS/WES, in that the technique can be used as a screening tool to identify unknown genetic aberrations across the whole genome/exome, a benefit which may prove paramount in identifying novel therapeutic targets and disease biomarkers.

#### 4.2.2. The JAK-STAT Pathway

The JAK-STAT pathway is constitutively activated in 50% of myeloma samples as well as a proportion of surrounding bone marrow stromal cells [92, 93]. The principal method thought to induce JAK-STAT activation is through autocrine and paracrine stimulation with IL-6, a cytokine shown to be important in myelomagenesis through the regulation of growth and survival [94, 95]. One key consequence of JAK-STAT activation is overactivity of STAT3, a STAT family transcription factor which results in high expression of the antiapoptotic protein Bcl-x_L_ [94], a protein correlated to chemoresistance in myeloma patients [96]. With this, inhibition of STAT3 with compounds such as curcumin, atiprimod, and the JAK2 kinase inhibitor AG490 are associated with inhibition of IL-6-induced myeloma survival* in vitro* [97–99]. Furthermore, inhibition of STAT3 has been shown to sensitize the U266 myeloma cell line to apoptosis induced through conventional chemotherapy agents [100]. Development of STAT3 inhibitors may therefore facilitate improved results with conventional chemotherapy agents in the future.

#### 4.2.3. The Phosphatidylinositol-3 Kinase (PI3K) Pathway

A range of molecular signals, such as IL-6 and IGF-1, acting on tyrosine kinase receptors can activate the PI3K pathway leading to phosphorylation of the serine-threonine-specific kinase AKT. AKT then subsequently activates several downstream targets including mTOR, GSK-3B and FKHR which influence many processes including cell proliferation and apoptosis resistance. Deregulation of the PI3K pathway is thought to be important in myeloma as phosphorylated AKT, an indicative marker of pathway activity, is observed in approximately 50% of cases [101]. Additionally, DEPTOR, a positive regulator of the PI3K pathway is commonly upregulated in myeloma, especially in those with* MAF* translocations [102], further demonstrating pathway activity. Of interest, unlike the MAPK pathway, the PI3K pathway is rarely mutated in myeloma [5]. However, as the pathway is known to be active, therapeutic targeting of PI3K is of interest within myeloma research.

### 4.3. Cell Cycle Deregulation

Alongside the overexpression of cyclin D genes in myeloma, the loss of function to negative cell cycle regulatory genes also proves to be a key event which destabilises cell cycle regulation. For example, the downregulation of* CDKN2C* through del(1p), or the inactivation of* CDKN2A* via DNA methylation changes may both deregulate the G_1_/S transition as these genes encode cyclin-dependent kinase inhibitors [65, 103]. Inactivation of the tumour suppressor gene* RB1*, a negative cell cycle regulator, also affects the G_1_/S transition and may occur frequently due to monosomy 13 or infrequently due to homozygous deletion or mutational inactivation [57]. The disruption of* RB1* is known to be a key pathological event in many cancers and the development of anti-cancer drugs targeting cell cycle regulators, including* RB1*, is a rapidly growing field.

### 4.4. Abnormal DNA Repair

Chromosomal instability is a defining feature of myeloma and contributes to the perpetual accumulation of genetic aberrations during disease progression. Despite this, consistent mutations of DNA repair genes have not been demonstrated in the disease, with loss of* TP53* function through del(17p), found in 10% of cases, the most common finding. Another gene of emerging importance however is* PARP1*, a gene encoding the PARP1 enzyme which contributes to repairing ssDNA breaks. A recent GEP study has demonstrated that increased expression of* PARP1* is associated with shortened survival in myeloma patients [104], whereas another GEP study identified the gene as one of 15 which may be used as an expression signature to define high-risk disease [105]. Investigations in this area may prove pivotal for myeloma, as PARP inhibitors have shown promising activity in clinical trials [106]. This activity is especially prominent in cancers with defective homologous recombination (HR)-mediated DNA repair mechanisms, as cells with defects in this system are sensitized to PARP inhibitors [107]. Although not a recognised* de novo* finding for myeloma, it has been shown that bortezomib can induce a HR-mediated DNA repair defective state, a so called “BRCAness”, through interference of BRCA1 and RAD51 recruitment to the sites of dsDNA breaks* in vitro* [104]. Thus, bortezomib and a PARP inhibitor may induce synthetic lethality and be utilised as a future combined treatment for myeloma.

### 4.5. Abnormal RNA Editing

A recent study revealed that nearly half of 38 myeloma samples contained mutations in genes involved in RNA processing, protein translation and the unfolded protein response (UPR) [5]. Four different mutations of* DIS3*, a gene encoding an exonuclease serving as the catalytic component of the exosome complex involved in regulating the abundance of RNA species [108, 109], were observed in 11% of samples [5]. The* DIS3* gene has been mapped to 13q22.1, and in three out the four mutations identified loss of function was exhibited by monoallelic mutation coupled to deletion of the remaining allele [5]. This demonstrates the key contribution del(13) is likely to play to* DIS3* mutations and suggests another implication of this genetic aberration alongside* RB1* haploinsufficiency. Furthermore, two of the four mutations in* DIS3* have been functionally characterised in microorganisms where they result in loss of enzymatic activity with consequential accumulation of RNA targets [110, 111]. As it has been shown that the exosome plays a vital role in regulating the available pool of mRNAs for translation [112], these mutational findings indicate that loss of* DIS3* activity may contribute to myelomagenesis through deregulation of protein translation. Another gene,* FAM46C*, implicated in del(1p) and previously discussed, gives further support for the role of translational control in myeloma pathogenesis, as WGS/WES found this gene to be mutated in 13% of samples [5]. This frequency supports the implication of* FAM46C* in myeloma pathogenesis as recurrently mutated genes are likely to be of biological significance.

### 4.6. Protein Homeostasis: The Unfolded Protein Response

The UPR is essential for the normal functioning of plasma cells as it serves a critical function in the efficient production of immunoglobulin by regulating cellular responses to unfolded/misfolded protein in the endoplasmic reticulum. Of interest, sequencing has revealed mutations of the* LRRK2* gene at a frequency of 8% [5].* LRRK2* encodes a serine-threonine kinase responsible for phosphorylating the eukaryotic translation initiation factor 4E-binding protein 1 (EIF4EBP1), a protein which functions in regulating protein translation.* LRRK2* is predominantly known for its association with Parkinson's disease, where mutations in the gene are linked to a predisposition for the condition [113, 114]. As with other neurodegenerative conditions, Parkinson's disease is in part characterised by a dysfunctional UPR and abrogated protein, and as myeloma has a vastly increased rate of immunoglobulin production [115, 116], any changes in protein homeostasis are likely to be pathogenically important. Of related interest, sequencing data has also revealed mutations in the UPR gene* XBP1*, although at a low frequency of 3% [5]. When over-expressed in transgenic mice, a splice form of* XBP1* has been shown to induce a myeloma-like syndrome [117], whereas in mice deficient of* XBP1* B cells are able to proliferate and construct germinal centres but are unable to differentiate into immunoglobulin secreting plasma cells [118]. The exact role of* XBP1* in human myeloma pathogenesis is unclear, although a recent study has shown that finding a high ratio between un-spliced and spliced variants of* XBP1* in myeloma samples is linked to a poor outcome and serves as an independent prognostic factor [119].

### 4.7. Abnormal Plasma Cell Differentiation

One method to establish biologically significant gain-of-function changes in cancer genomes is to use WGS/WES to search for recurrent identical mutations in candidate oncogenes. Utilising this method, Chapman et al. found two myeloma patients from 38 harboured an identical mutation (K123R) in the DNA-binding domain of the interferon regulatory factor 4 (*IRF4*) [5]. As its name suggests,* IRF4* is involved in regulating the transcription of interferon's whilst it also plays an important role in B cell proliferation and differentiation. Interestingly, a recent RNA-inference-based genetic screen revealed that* IRF4* function is required for myeloma cell line survival as inhibition of the gene proved toxic to the malignant cells [120], an* in vitro* finding supporting the genes role in pathogenesis. One way in which* IRF4* acts is as a transcription factor for* BLIMP1*, also a transcription factor itself which plays a key role in plasma cell differentiation. The Chapman et al. study identified two mutations in the* BLIMP1* gene from their 38 samples, and as loss-of-function mutations in* BLIMP1* are known to occur in diffuse large B-cell lymphoma [121], this suggests* BLIMP1* mutations may be of pathogenic importance to myeloma. Of consideration, as myeloma is a malignancy of terminally differentiated plasma cells, the importance of dysfunction within differentiation pathways may be of less importance than in cancers of immature cells, further studies are however required to investigate this.

### 4.8. Myeloma Bone Disease

Bone disease occurs in 80–90% of patients with myeloma and can be either focal or diffuse resulting in pain, pathological factures, cord compression and hypercalcaemia. A recent GEP study aimed to identify the molecular basis of patients presenting with bone disease in order to elucidate whether a gene expression signature could identify those at high-risk of skeletal-related events after randomization into one of two bisphosphonate arms [122]. The study identified that 50 genes were significantly associated with presenting bone disease, mostly from pathways involved in growth factor signalling, apoptosis and transcription regulation. The two most significantly differently expressed genes were the Wnt pathway inhibitors* DKK1* and* FRZB*. The Wnt pathway is known to be important in regulating bone turnover and* DKK1* has been shown to both inhibit osteoblast differentiation and increase bone resorption through an increase in the RANKL/OPG ratio [123, 124]. An antibody against DKK1 is now being tested in clinical trials after showing promise by improving bone disease and inhibiting myeloma cell growth in a murine model [125]. The GEP study was also able to make more generalised observations of which some have been previously been reported [72, 126]. For example, bone disease is more prevalent in those with a hyperdiploidy signature and less associated with *t*(4; 14) and* MAF* translocations. Interestingly, this study found that* DKK1* and* FRZB* were more highly expressed in hyperdiploid tumours, providing a potential explanation for this finding. Secondly, patients with bone disease have shorter OS compared to those without [127], a finding which suggests bone disease significantly contributes to the impaired outcome of these patients, or, alternatively, that disease biology in myeloma with bone disease is distinctly different.

## 5. Epigenetic Changes in Myeloma

**Figure 5:**
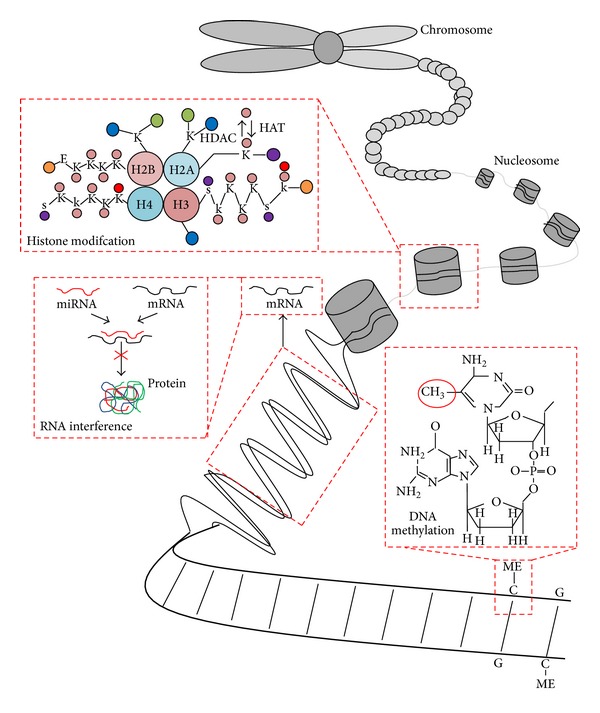
*Mechanisms of epigenetic regulation*. Three main forms of epigenetic modification include histone modification, RNA interference, and DNA methylation. Histone (chromatin) modification refers to the covalent posttranslational modifications to the N-terminal tails of the four core histone proteins; this modification is commonly acetylation/deacetylation changes at lysine residues mediated by histone acetyltransferases (HATs) and histone deacetylases (HDACs). RNA interference is predominantly mediated through microRNAs, which inhibit the translation of mRNA into protein. DNA methylation occurs at cytosine residues of CpG dinucleotides and acts to regulate gene expression. Pink circle = acetyl group, purple circle = phosphate group, red circle = methyl group, blue circle = carboxyl terminus, green circle = ubiquitin, orange circle = amino terminus, k = lysine, E = glutamic acid, S = serine. H2A, histone 2A; H2B, histone 2B; H3, histone 3; H4, histone 4.

The study of epigenetics is an emerging field in myeloma and one which is demonstrating an increasing amount of influence on pathogenesis [128]. As outlined in Figure 5, the three main areas of epigenetic regulation include histone modification, RNA interference and DNA methylation.

### 5.1. DNA Methylation

DNA Methylation changes occur at CpG dinucleotides which are generally found at higher frequencies in promoter regions, repeat sequences and transposable elements. Changes in DNA methylation act to regulate gene expression and are known to be important in contributing to cell development and differentiation as well as the progression of many cancers. Myeloma genomes, as for many other cancers, often follow a recognised pattern of methylation represented by global DNA hypomethylation and gene-specific hypermethylation [129]. A recent study using a genome-wide methylation microarray built on this knowledge to demonstrate that a marked loss methylation occurred at the transition from MGUS to myeloma [129]. Furthermore, gene-specific hypo and hypermethylation was demonstrated at this transition with the genes affected involved in the cell cycle, transcriptional and cell development pathways [129]. During progression from myeloma to PCL, rather than finding global DNA hypomehtylation, gene-specific hypermethylation was found in genes involved in cell adhesion and cell signalling [129]. This finding suggests these methylation changes may contribute to destabilisation of the stromal-clone relationship and promotion of clonal transition into the circulation and a proliferating leukaemic phase. The most significant DNA methylation changes, influencing cell survival, cell cycle progression and DNA repair, are seen in *t*(4; 14) tumours [33, 130], presumably as they over-express the* MMSET* gene which encodes a HMT transcription repressor.

### 5.2. Histone Modification

Other genes involved in methylation and chromatin modification are also deregulated in myeloma, including* KDM6A*,* MLL* genes and* HOXA9* [5]. Recent sequencing observed that* HOXA9* was ubiquitously expressed across 38 myeloma samples and hypothesised whether this gene represented a candidate oncogene [5]. The* HOXA9* gene is primarily regulated by HMTs and encodes a DNA-binding transcription factor which contributes to regulating gene expression, morphogenesis and differentiation. As the majority of cases over-expressing* HOXA9* in the study exhibited bi-allelic expression, consistent with deregulation of an upstream HMT event, genes involved in regulating* HOXA9* were evaluated for mutations with findings revealing mutations in several genes:* MLL*,* MLL2*,* MLL3*, and* MMSET* [5]. To establish the functional importance of* HOXA9* expression in myeloma, gene knockdown studies in a range of myeloma cell lines was performed and demonstrated that* HOXA9*-depleted cells incurred a competitive disadvantage against those with remaining* HOXA9* function [5]. These findings indicate that the expression of* HOXA9* has a role in myeloma pathogenesis and that these epigenetic changes may represent new therapeutic targets in myeloma.

### 5.3. MicroRNA Changes

MicroRNA (miRNA) genes encode a class of small RNAs (17–25 base pairs) which function to regulate the translation of other proteins by forming complementary base parings to specific mRNA transcripts. Studies have shown that miRNAs can act as both tumour suppressors and oncogenes in a range of cancers and that their transcriptional control is regulated by promoter methylation changes [131]. A substantial amount of work has been completed to investigate which miRNAs are differentially expressed in myeloma [132–134], and it has been shown that miRNA changes can deregulate genes and pathways relevant to myeloma pathogenesis including cell cycle progression,* TP53* and* MYC* [135–137]. Although there is some discrepancy between which miRNAs are differentially expressed, and when, in myeloma, the overall conclusion is that miRNA deregulation is likely to be an important contributor to the malignancy and that further investigation is warranted to improve understanding and to highlight potential treatment targets.

## 6. Conclusion

Myeloma is a highly heterogeneous disease and one which may progress from an indolent, asymptomatic phase, to an aggressive extramedullary phase as genetic “hits” are acquired over time. The primary genetic events contributing towards plasma cell immortalisation can be broadly divided into a hyperdiploid group, characterised by trisomies of odd numbered chromosomes, and a nonhyperdiploid group, characterised by IGH@ translocations to various partner chromosomes; it appears that overexpression of the cyclin D family of genes is an almost universal sequelae of primary events. Secondary genetic events, contributing to disease progression, are complex and involve secondary translocations, CNVs, acquired mutations, LOH, and epigenetic modification. From the use of molecular techniques to investigate myeloma, many of these primary and secondary events are now well characterised, and from this characterisation, it is apparent that disease behaviour can be correlated to the genetic makeup of a patient's disease. With this, it is therefore essential that genetic research remains focused in translating molecular characterisation into clinically useful advances.

## Figures and Tables

**Table 1:** Diagnostic criteria for myeloma of undetermined significance (MGUS), smouldering multiple myeloma (SMM), myeloma, and plasma cell leukemia (PCL). Reproduced from international myeloma working group, 2003 [37].

MGUS	SMM	Myeloma	PCL
Serum M-protein <30 g/L	Serum M-protein ≥30 g/L **AND/OR** Bone marrow clonal plasma cells ≥10%	M-protein in serum and/or urine.* No specific concentration required	Presence of ≥20% circulating plasma cells Absolute level of >2.0 × 10^9^/L
Bone marrow clonal plasma cells <10%. **If done**—low level of plasma cell infiltration in a trephine biopsy		Confirmed clonal plasma cells in bone marrow	
Absence of end-organ disease and symptoms	Absence of end-organ disease and symptoms	Presence of myeloma-related organ or tissue impairment (ROTI)**	

*1-2% of patients have no detectable M-protein in serum or urine but do have myeloma-related organ or tissue impairment (ROTI) and increased intramedullary plasma cells; this is termed nonsecretory myeloma. **ROTI: corrected serum calcium >0.25 mmol/L above the upper limit of normal or >2.75 mmol/L, creatinine >173 mmol/L, Hb 2 g/dL below the lower limit of normal or <10 g/dL, lytic bone lesions or osteoporosis with compression fractures (may be clarified by CT or MRI), symptomatic hyperviscosity, amyloidosis, recurrent bacterial infections (>2 episodes in 12 months).
